# A Systematic Review and Meta-Analysis of Randomized Controlled Trials on Supine vs. Nonsupine Endotracheal Intubation

**DOI:** 10.1155/2023/5496368

**Published:** 2023-07-06

**Authors:** Chriselyn F. Palma, Radwan Mashina, Claire Chen, Tareq Arar, Marwan Mashina, Yussef Al Ghoul, Banreet Dhindsa, Rajany Dy

**Affiliations:** ^1^University of Las Vegas Nevada School of Medicine, 1707 W. Charleston Blvd Suite, 230 Las Vegas, NV 89102, USA; ^2^Jordan University of Science and Technology, 3030 Ar-Ramtha, Jordan; ^3^Medstar Washington, 110 Irving St., NW Washington, D.C. 20010, USA; ^4^University of Florida, 1600 SW Archer Rd, Gainesville, FL 32608, USA; ^5^University at Buffalo, Erie County Medical Center, David K. Miller Building, 462 Grider St., Buffalo, NY 14215, USA; ^6^University of Nebraska Medical Center, 983332 Nebraska Medical Center, Omaha, NE 68198-3332, USA

## Abstract

**Background:**

This systematic review and meta-analysis of randomized controlled trials (RCTs) was performed to compare the safety and efficacy of supine vs. nonsupine positions during intubation.

**Methods:**

Based on the literature from inception to October 2020, 13 studies with nonemergent intubation in supine and nonsupine positions were chosen using PRISMA and MOOSE protocols. Pooled estimates were calculated using random-effects models with 95% confidence interval (CI). The primary outcome was a successful intubation, attempt, and duration of intubation. The secondary outcome was adverse events (trauma and hypoxia). Bias was evaluated qualitatively, by visual analysis, and quantitatively through the Egger test.

**Results:**

The final analysis included 13 clinical trials with 1,916 patients. The pooled success rates in the supine vs. lateral positions were 99.21% and 98.82%. The supine vs. semierect positions were 99.21% and 98.82%. The 1st attempt success rate in the supine vs. lateral position was 85.35% and 88.56% compared to 91.38% and 90.76% for the supine vs. semierect position. The rate of total adverse events in the supine position was 3.73% vs. 6.74% in the lateral position, and the rate of total adverse events in the supine position was 0.44% vs. 0.93% in semierect position. Low to substantial heterogeneity was noted in our analysis. *Discussion*. There is no significant difference between total successful intubations and success from 1st intubation attempt between supine and nonsupine positions. However, there are slightly higher rates of adverse events in nonsupine position. Addition of more recent studies on supine vs. nonsupine intubations would improve this study. Given these findings, it is important to develop more studies regarding different intubation positions and techniques with the aim of improving efficacy and decreasing adverse outcomes. *Other*. This review is not registered in a public database. This research did not receive any specific grant from funding agencies in the public, commercial, or not-for-profit sectors.

## 1. Introduction

Endotracheal intubation is a procedure performed to secure the airway for various elective and emergent indications, including surgeries, respiratory failure, and altered mental status [1]. The first reported endotracheal intubation was performed in 1878 by William Hack to remove vocal cord polyps [2]. Multiple enhancements have been applied to this procedure to improve outcomes and reduce adverse events [3]. Success in the first attempt and hypoxia are well-determined factors for adverse events related to endotracheal intubation.

There are currently no guidelines regarding optimum positioning during endotracheal intubation. The supine position is the most common position used for endotracheal intubation [4]. With the advent of medicine, other positions, including lateral and semierect, are being used for various indications [5, 6]. Experience in intubation in nonsupine positions is vital as inadequate airway management can be catastrophic, leading to hypoxia, brain injury, and even death [7]. Intubation in a nonsupine position, especially in the lateral position, could be challenging for many reasons, such as unfamiliarity and lack of experience between providers and distortion of the upper airway anatomy.

Multiple studies have suggested that intubation in the semierect position can improve the upper airway's alignment by providing a better glottic view and reducing the time of successful intubation [8–10]. It is hypothesized that intubation in the semierect position can lead to increased functional residual capacity and duration of nonhypoxic apnea compared to the supine position [11–14]. The lateral position during intubation is needed in many situations: patients with back pain/lesions, increased aspiration risk, dislodgement of the endotracheal tube during surgery in the lateral position, amongst various other indications [15–17].

This study was intended to do a systematic review and meta-analysis of randomized controlled trials (RCTs) to compare the safety and efficacy of semierect vs. supine and lateral vs. supine positioning during endotracheal intubation.

## 2. Materials and Methods

### 2.1. Search Strategy

**Figure 1:**
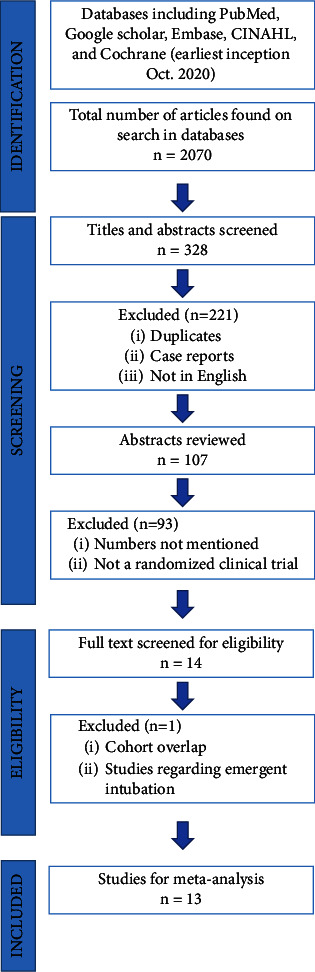
Literature search using PRISMA and MOOSE protocol.

A comprehensive search of several databases and conference proceedings, including PubMed, Google Scholar, Embase, CINAHL, and Cochrane (earliest inception to October 2020), was carried out to identify RCTs comparing endotracheal intubation in the supine vs. semierect position and supine vs. lateral position. We performed the search according to the Preferred Reporting Items for Systematic Reviews and Meta-Analysis (PRISMA) guidelines and Meta-Analysis of Observational Studies in Epidemiology (MOOSE) protocol (Figure 1).

We searched the abovementioned databases using a combination of keywords “intubation,” “supine,” “lateral,” “ramped-up,” “semi-erect,” and “complications.” Two authors reviewed study titles and abstracts and excluded studies not related to the research question according to semidetermined inclusion and exclusion criteria. The remaining articles were read in detail to find studies with relevant information. Conflicts in the final inclusion of a study were resolved by discussion with a third author. The authors reviewed references of the selected articles and other relevant articles, and the appropriate studies were added.

### 2.2. Study Selection

In this meta-analysis, we included RCTs comparing the safety and technical success of endotracheal intubation in the supine vs. semierect position and supine vs. lateral position.

We applied the following exclusion criteria in our study: (1) all studies other than randomized clinical trials, (2) studies with fewer than 50 patients, (3) studies with patients undergoing emergent endotracheal intubation, (4) studies performed in pediatric populations (age <18 years), and (5) studies not published in the English language.

In the case of cohort overlap or multiple publications from a single cohort, data from the most recent and most relevant articles were included.

### 2.3. Data Abstraction and Quality Assessment

Three authors reviewed the selected studies and collected data related to study outcomes in a standardized form. Two authors independently performed quality scoring. We used the Oxford JADAD score for evaluation of the quality of our study. This quality score consisted of 3 aspects, as shown in Table 1.

### 2.4. Outcomes Assessed

Primary outcomes are as follows:
  1. Pooled rate of total successful intubations: supine vs. lateral position and supine vs. semierect position
  2. Pooled rate of success from 1st, 2nd, and 3rd intubation attempts: supine vs. lateral position and supine vs. semierect position
  3. Pooled rate of time for successful intubation: supine vs. lateral position and supine vs. semierect position
Secondary outcomes are as follows:
  1. Pooled rate of total and individual adverse events: supine vs. lateral and supine vs. semierect positions
  2. Correlation of Mallampati score to successful intubation

#### 2.4.1. Assessment Methodology and Definitions

We matched the data collected between all the study groups (supine position, lateral position, and semierect position) before sending it for statistical analysis.

The semierect position was defined as raising the back by 25° from horizontal plane in 2 studies, the elevation of the upper body until an imaginary horizontal line can be drawn between the sternal notch space and the external ear in one study, and as raising the table's back-end to the patient's comfort level [5, 21, 24, 29].

Definition of outcomes are as follows:

  Successful intubation was defined as the appearance of end-tidal carbon dioxide on capnography [5, 18, 21, 24, 25, 27–29].
  Failed intubation was defined as a failed 3rd attempt in four studies [5, 24, 25, 29], failed 4th attempt in two studies [18, 22], and failed 5th attempt in one study [23]. In two studies, it was defined as the inability to complete intubation within 3 minutes of cessation of preoxygenation [27, 28].
  A failed attempt was defined in only five studies. Definitions were attempts to advance the tube that took more than 30 seconds [18], failure after four adjusting maneuvers [23], no positive capnography for three breaths following an intubation attempt [5], failure to finish attempt within 60 seconds [18], and attempts taking more than 120 seconds [26].
  Desaturation was defined as oxygen saturation on pulse oximetry less than 95% in four studies [24–26, 29], less than 92% in three studies [5, 27, 28], and 90% in two studies [18, 20].

### 2.5. Statistical Analysis

To measure the individual pooled estimates, we utilized meta-analysis techniques, as suggested by DerSimonian and Laird, using the random-effects model. If an incidence of an outcome was zero, we performed statistical analysis after adding a continuity correction of 0.5 to the number of incident cases. We performed a heterogeneity assessment of study-specific estimates by utilizing the Cochran *Q* statistical test for heterogeneity, 95% prediction interval (PI), which addresses the effects' dispersion, and the *I*^2^ statistics. This test reported heterogeneity as low, moderate, substantial, and considerable based on values of <30%, 30–60%, 61–75%, and >75%, respectively. We evaluated publication bias both qualitatively, by visual analysis of funnel plot, and quantitatively, by the Egger test. In the case of publication bias, we performed additional analysis using the fail-safe *N* test and Duval and Tweedie's “trim and fill” test to determine the impact of the bias. The degree of publication bias impact was classified into three levels based on the similarity between the reported results and estimated results if no bias. The impact was classified as minimal if both results were estimated to be the same, modest if changed effect size substantially but the conclusion remained the same, and severe if bias compromised the final findings of the analysis.

We performed all analyses using comprehensive meta-analysis software, version 3 (BioStat, Englewood, NJ).

## 3. Results

### 3.1. Search Results and Population Characteristics

The search yielded 13 studies comparing intubation in the supine position vs. other nonsupine positions. Two studies with cohort overlap were found, and one was excluded based on the relevance of the trial and information available within the manuscript. Overall, 9 studies provided data on supine vs. lateral positioning and 4 studies provided data on the supine vs. semierect position.

We followed PRISMA guidelines for study selection as illustrated in Figure 1.

Population characteristics were similar between the study groups, as illustrated in Tables 2 and 3.

### 3.2. Characteristics and Quality of Included Studies

All 13 studies were RCTs. Eight studies were single-center and five were multicenter. There were no population-based studies. All studies commented sufficiently on clinical outcomes and variables under the study, including the intubation method, side of lateral position, and degree of semierect position, as summarized in Tables 4 and 5. The studies included were not double blinded due to the nature of the procedure under investigation, as it is difficult to blind the investigator from the patient's position during intubation. Based on the JADAD score provided in Table 1, 8 studies were considered high quality and five studies were low quality.

### 3.3. Meta-Analysis Outcomes

#### 3.3.1. Primary Outcomes

**Figure 2:**
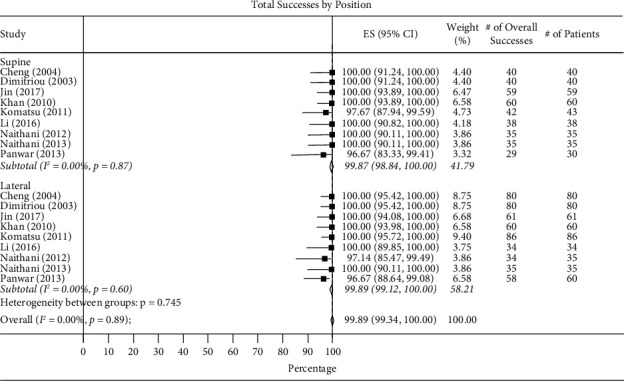
Total success by supine vs. lateral positions.

**Figure 3:**
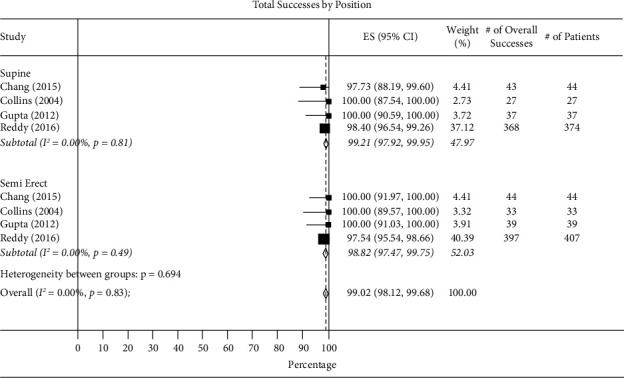
Total success by supine vs. semierect positions.

*(1) Total Success Rate of Intubation*. Total intubation success rate in supine vs. lateral positions was 99.87% (95% CI 98.84–100.00, *I*^2^ = 0.00%) and 98.89% (95% CI 99.12–100.00, *I*^2^ = 0.00%) (Figure 2) and in the supine vs. semierect position was 99.21% (95% CI 97.92–99.95, *I*^2^ = 0.00%) and 98.82% (95% CI 97.47–99.75, *I*^2^ = 0.00%) (Figure 3).

**Figure 4:**
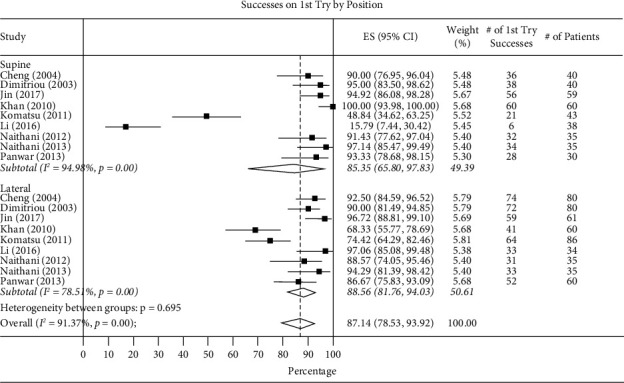
Total success on first try by supine vs. lateral positions.

*(2) The Success Rate from the 1st, 2nd, and 3rd Attempts*. In studies comparing supine vs. lateral positions, rates of success from the first, second, and third attempts in the supine position were 85.35% (95% CI 65.80–97.83, *I*^2^ = 0), 99.07% (95% CI 84.50–100, *I*^2^ = 43.05%), and 68.38% (95% CI 15.98–100), while the lateral position success rates were 88.56% (95% CI 81.76–94.03, *I*^2^ = 0), 90.85% (95% CI 76.01–99.67, *I*^2^ = 30.64), and 82.73% (95% CI 35.85–100, *I*^2^ = 23.21%). *P* values were significant for the first (*P* ≤ 0.001) and second (*P* = 0.05) attempts but not significant for the third attempt (*P* = 0.27) (Figure 4).

**Figure 5:**
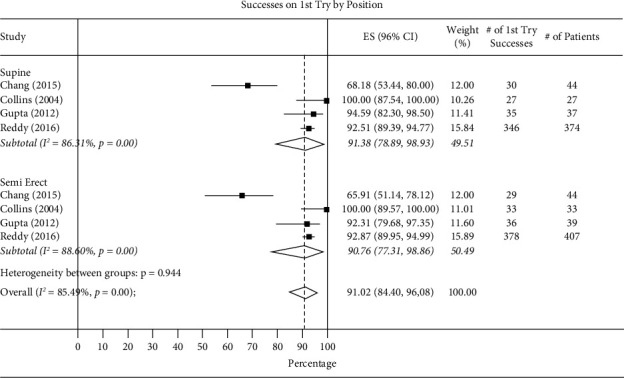
Total success on first try by supine vs. semierect positions.

In studies comparing supine vs. semerect positions, rates of success from the first, second, and third attempts in the supine position were 91.38% (95% CI 78.89–98.93, *I*^2^ = 88.31%), 80.83 (95% CI 65.21–93.36), and 20.26 (95% CI 0.07-63-84), while in the semierect position rates were 90.76% (95% CI 77.31–98.86, *I*^2^ = 88.60%), 75.09 (95% CI 54.94–91.49), and 26.12 (95% CI 3.86–55.54). *P* values were *P* ≤ 0.001 for the first attempt, *P* = 0.54 for the second attempt, and *P* ≤ 0.001 for the third attempt (Figure 5).

*(3) Intubation Duration*. In supine vs. lateral positions, supine intubation duration averaged 37.61 ± 35.79 seconds while lateral intubation averaged 30.26 ± 10.67 seconds. In supine vs. semierect positions, supine intubation averaged 29.17 ± 6.76 seconds while semierect intubation averaged 25.81 ± 8.26 seconds.

#### 3.3.2. Secondary Outcomes

*(1) Rate of Adverse Events*. In the supine vs. lateral position, the rate of total adverse events in supine position was 3.73% (95% CI 0.51–8.91, *I*^2^ = 64.93%) and in the lateral position was 6.74% (95% CI 2.03–13.45, *I*^2^ = 77.90%). *P* value was statistically significant (*P* ≤ 0.001). Complications mentioned in the studies were esophageal intubation, mucosal trauma, including lip or dental injury and oropharyngeal bleeding, sore throat, cough, dryness of mouth, hoarseness, and dysrhythmias (PVCs and PACs). Based on these reported complications, the rate of esophageal intubation in the supine position was 1.07% (95% CI 0.02–3.10, *I*^2^ = 6.16%) and in the lateral position was 1.65% (95% CI 0.08–4.45, *I*^2^ = 52.41%). *P* value was not statistically significant (*P* = 0.12). The rate of mucosal injury in the supine position was 0.17% (95% CI 0.00–1.49, *I*^2^ = 0.00%) and in the lateral position was 0.39% (95% CI 0.00–1.64, *I*^2^ = 4.17%) with *P* = 0.68.

In the supine vs. semierect position, the adverse events mentioned were hypoxia and regurgitation. The rate of total adverse events in the supine position was 0.44% (95% CI 0.00–4.58, *I*^2^ = 72.96%) and in the semierect position was 0.93% (95% CI 0.00–7.07, *I*^2^ = 83.59%). *P* value was statistically significant (*P* ≤ 0.001). The rate of hypoxia in the supine position was 0.17% (95% CI 0.00–2.98, *I*^2^ = 61.02%) and in the semierect position was 0.42% (95% CI 0.00–1.61, *I*^2^ = 73.06%). *P* value was significant (*P* = 0.01).

*(2) Correlation of Mallampati Score with Adverse Events*. In the supine vs. lateral position, there was a positive correlation between increased Mallampati score and rate of adverse events in both positions. However, it is not possible to analyze the correlation with the supine vs. semierect position because the majority of the studies did not investigate adverse events.

### 3.4. Validation of Meta-Analysis

#### 3.4.1. Sensitivity Analysis

To determine if any individual study had an influential effect on the meta-analysis, we sequentially excluded a study each time and analyzed the impact on the primary outcomes. The outcomes and heterogeneity of the meta-analysis were not predominantly affected by a single study.

#### 3.4.2. Heterogeneity

We evaluated the dispersion of analysis outcomes using *I*^2^ percentage values, which determine whether the dispersion is true vs. chance. The *I*^2^ test reported heterogeneity as low, moderate, substantial, and considerable based on values of <30%, 30–60%, 61–75%, and >75%, respectively.

## 4. Discussion

Our study is the first meta-analysis of RCTs comparing supine, lateral, and semierect positions in endotracheal intubation. Data analysis showed no evidence of favorable outcomes for intubation in the supine position vs. lateral and semierect positions regarding total success rate; success from the first, second, and third attempts; and time required to complete intubation.

In comparing randomized-controlled trials for the supine vs. semierect position, the majority of the studies showed a comparable total success rate. However, the success rate from the first attempt in the supine vs. semierect position was noticeably lower in one study [18]. From reviewing the different studies available that compared the supine vs. semierect position, experience was not a factor as anesthesiologists who participated in Chang's study were noted to be “two experienced anesthesiologists.” Though Chang et al. did not mention the number of years to quantify experience, other studies, such as the study by Reddy et al. involved anesthesiologists with varying years of experience who had slightly more successful first attempt intubations with the semierect position (92.4 vs 92.9%) in a larger sample size. In addition, the use of the Mallampati score vs. the Cormack–Lehane grade did not show the study was skewed on difficult airways. Despite this information, there are several notable differences that could contribute to the difference in this finding.

The first noticeable difference is the region where the study was conducted. The study by Chang et al. was performed in Korea, whereas studies by Collins, Gupta, and Reddy were carried out in western countries. The difference in the bone or cranial structures between eastern and western counterparts [30] could have contributed to a lower success rate in the first attempt of intubation. Given the differences in body metrics, it can also be considered that the BMI and the Mallampati score may not be adjusted appropriately as these studies were usually performed on western body types and could have underestimated the difficulty of intubation [31]. It is also important to note the differences in BMI among all four studies. Chang's study has the lowest BMI (23.85) compared to Reddy's (28), Gupta' (46.8), and Collins' (49.9). As Reddy's study has mentioned, the semierect position can improve the view on patients who will likely be more difficult to intubate. Therefore, the angle of 25° may not be the most optimal angle for patients in Chang's study given the differences mentioned.

Aside from the structure of the patient, there are also differences in the position, intubation method, and medication used that could account for the lower success rate on the first attempt of intubation. The degree of semierect positions in the studies was variable. Some semierect positions were measured in 25°, 30°, and 35°, while others were dependent on patient preference and the angle the EAM is horizontal to the sternal notch. The variability of the degrees of nonsupine positions may have affected the outcomes. It is possible that studies may not have optimized the view of intubation due to these differences. Compared to the other studies, Chang used fiberoptic bronchoscopy instead of laryngoscopy with a GlideScope to perform the procedures. For sedation, sevoflurane was used instead of fentanyl. Compared to the laryngoscope, the bronchoscope is a more flexible device. If the anesthesiologist is initially not comfortable with the technique, especially at a new angle, this adjustment can count for the increased number of tries for intubation. Given that only two anesthesiologists participated in the study, the technique could have improved after a few tries leading to more successful intubations. The method itself of intubating through a bronchoscope could also account for the increased attempts given the criteria of 60 seconds or desaturation of less than 90% as the bronchoscope is longer and more flexible than the laryngoscope which the other studies used. Lastly, the type of sedation can also be considered as to help with the relaxation to improve airway clearance.

Another difference worth noticing is that the rate of hypoxia in semierect patients' intubations was statistically significant. Though some studies hypothesized that the nonerect position can increase functional residual capacity and duration of nonhypoxic apnea events, the analysis shows that there was actually less rates of hypoxia in supine positions. A possible explanation is that the studies that had these findings were carried out with angles of 20°, 25°, or 30° [11–14]. One explanation could be that two studies with the highest BMIs, Collins' and Gupta°s, did not follow these recommendations. In addition, lack of familiarity with intubating in the supine positions could have contributed to this difference.

In RCTs comparing supine vs. lateral positions, the total success rate was comparable in all studies. Two studies [25, 26] showed a significantly lower success rate from the first attempt in the supine position. Similarities between the studies by Li et al. and Komatsu et al. include outcomes (more intubation attempts required in supine positioning), investigator characteristics (a small number of experienced anesthesiologists), and patient population characteristics (predominantly female in their early to mid-50s with average BMI 23). Both papers theorized that with supine positioning, gravity causes physiologic changes within oropharyngeal tissues that may hamper intubation [25, 26]. Under anesthesia, the tongue relaxes and obstructs the trachea, causing resistance to bronchoscopic advancement. In addition, gravity causes the diameter between the epiglottis and posterior pharyngeal wall to decrease, causing the laryngoscopic blade to advance into the vallecula instead of the glottis. These obstructions may require the anesthesiologist or an assistant to reposition the mandible, increasing risk of hemodynamic instability in vulnerable patients.

However, key differences exist. Li et al. found that lateral positioning resulted in shorter time for intubation while Komatsu et al. found that time to intubation, intubation success, and airway complications were not significantly different. These differences in outcomes may be attributed to study design dissimilarities. The degree of the lateral positions was not provided though 7 studies mentioned that they maintained an axial position for the lateral patients. Though there is less variability compared to the semierect positioning, this difference might have affected the outcomes of the study. In addition, Li used a flexible fiberoptic bronchoscope while Komatsu used a novel video laryngoscope called the airway scope (AWS) that allows for improved visualization of the glottic opening. To our knowledge, there are no randomized control trials comparing the efficacy of flexible fiberoptic bronchoscopes compared to the AWS. Suzuki has described one case report of the AWS used in a morbidly obese patient with a full stomach in lieu of flexible bronchoscope [32], but this patient does not match the patient population of either studies, as their population's average BMI was approximately 23. Other studies have compared the AWS to C-MAC, GlideScope, and Macintosh laryngoscope and found that all three instruments had comparable first intubation attempt success rates, but the AWS had the best time to intubation, ease of intubation and grade 1 laryngeal view [33]. Given that the AWS had differences in performance metrics to the C-MAC and GlideScope, it is possible that the AWS performs differently from the flexible fiberoptic scope.

Notably, Komatsu et al. chose to have one experienced anesthesiologist assigned to laryngoscopy with a Macintosh #3 blade before intubation while the AWS was assigned to a different anesthesiologist. The investigator that performed laryngoscopy had limited experience, having only performed 5 with the Macintosh laryngoscope. Conversely, the investigator that performed the intubation was very experienced with the AWS, having performed 100 intubations using the AWS. Laterally positioned patients' bodies (but not heads and necks) were also stabilized with an assistant. Having multiple unblinded participants may have led to some indiscriminate bias, limiting true comparison between studies. In addition, the two studies differed in patient population as Komatsu excluded patients with ASA 4 or more and anticipated airway difficulties (Mallampati grade 4 and thyromental distance <6 cm) compared to Li's exclusion of patients with ASA 3 or more and any history of poor cardiopulmonary function. Komatsu's sicker population may have rendered intubation outcomes in different positions null.

The rate of total adverse events in both lateral and semierect positions was almost two times that with the supine position, (6.74% vs. 3.73%, *P* ≤ 0.001) and (0.93% vs. 0.44%, *P* ≤ 0.001), respectively. Our results suggest that while intubation in supine, lateral, and semierect positions is technically comparable, nonsupine positions might be associated with more adverse events. This result could be attributed to various reasons, including unfamiliarity and lack of experience between providers, distortion of the upper airway anatomy as occurs in the lateral position, and other unidentified variables [34, 35]. It is reasonable to think that as these positions are used more frequently, the rate of adverse events will become comparable to the supine position. However, it is important to be aware of the risk factors for adverse events during intubation.

A higher Mallampati score was associated with a higher rate of adverse effects in supine and lateral positions. This score is an independent risk factor for difficult intubation which is associated with multiple adverse events including hypoxia and traumatic intubations [36–38]. Older age correlated with an increased rate of adverse events in all positions and decreased rate of total success in supine and semierect positions. This finding is likely related to reduce upper airway size and increased Mallampati score and laryngoscopy grade in older population [39, 40]. Although male sex is associated with higher Mallampati score [40], upper airway collapsibility [39], and intubation forces [41], our analysis showed higher percentage of males correlated with increased rate of successful intubation and reduced rate of adverse events. Females have different upper airway anatomy, physiology, and biomechanics compared to males [39, 41–43]. Furthermore, in obese females, intubation is further complicated by impediment of laryngoscopy by patients' breasts [44]. Hasanin conducted a trial that showed modified-ramped position for females resulted in better outcomes compared to the classical ramped position [45].

Despite the different findings noted, it is important to mention the limitations of this study. One would be intubation techniques, methods, and positions were not compared. These differences could affect the accuracy of the analysis given the difference in performance metrics. Another is the different levels of experience anesthesiologists have per study. However, the information on intubations per level of experience was not discussed in all of the studies, so a proper comparison could not be achieved. Finally, the studies had different definitions of failed intubations, specifically number of attempts or time period. The definitions for failed attempts with time periods also vary. The different criteria for failed intubation were not statistically analyzed, which makes it another limitation of this study.

In conclusion, there are no significant differences regarding success rates, attempts, and time for intubation with supine vs. lateral vs. semierect positions. However, there are slightly higher rates of adverse events in the semisupine position as compared to the supine position. A variety of factors, such as ethnicity, age, and sex, can affect the outcomes. It would be interesting to see if more recent data show any changes in primary and secondary outcomes based on the position and risk factors. Furthermore, studies can be carried out to address the limitations of this study. Lastly, more studies can be performed regarding different techniques to improve efficacy and decrease adverse outcomes of these variables.

## Figures and Tables

**Table 1:** Oxford JADAD score for quality scoring of RCTs selected.

Oxford JADAD score				
Study	Year	Randomization	Blinding	Withdrawals
*RCT comparing supine vs. lateral positions*				
Cheng et al. [22]	2004	1	0	1
Dimitriou et al. [23]	2003	1	0	1
Jin et al. [24]	2017	2	0	1
Khan et al. [5]	2010	2	0	1
Komatsu et al. [25]	2011	2	0	1
Li et al. [26]	2016	2	0	1
Naithani et al. [27]	2012	2	0	1
Naithani et al. [28]	2013	1	0	1
Panwar et al. [29]	2013	2	0	1

*RCT comparing supine vs. semierect positions*				
Chang et al. [18]	2015	2	0	1
Collins et al. [19]	2004	2	0	1
Gupta and Rusin [20]	2012	1	0	1
Reddy et al. [21]	2016	1	0	1

**Table 2:** Population characteristic in supine vs. lateral positions.

Supine vs. lateral positions						
Study	Location	Country	Number of patients	Age	Male (%)	BMI
Cheng et al. [22]	Single-center	Taiwan	40	40.6	52.5	23.96
			80	43.35	50.6	24.15

Dimitriou et al. [23]	Multicenter	Greece and Australia	40	46	45	24.84
			80	44	40	24.85

Jin et al. [24]	Multicenter	China	59	55.39	59.3	23.56
			61	55.08	60.6	22.53

Khan et al. [5]	Single-center	Pakistan	60	35.8	63.3	—
			60	35	63.3	—

Komatsu et al. [25]	Single-center	Japan	43	62	34.8	23
			86	56	31.3	22

Li et al. [26]	Multicenter	China	38	52.6	31.5	23.75
			34	50.5	35.2	24.68

Naithani et al. [27]	Single-center	India	35	30.8	—	21.28
			35	28.51	—	20.94

Naithani et al. [28]	Multicenter	India	35	29.51	—	19.19
			35	31.14	—	20.45

Panwar et al. [29]	Single-center	India	30	—	33.3	22.46
			60	—	26.6	22.48

**Table 3:** Population characteristic in supine vs. semierect positions.

Supine vs. semierect positions						
Study	Location	Country	Number of patients	Age	Male (%)	BMI
Chang et al. [18]	Single-center	Korea	44	48	47.7	23.52
			44	49	45.4	23.85

Collins et al. [19]	Multicenter	Philadelphia	27	43.3	7.40	46.9
			33	41.9	18.18	49.9

Gupta and Rusin [20]	Multicenter	US	37	—	—	51.6
			39	—	—	46.8

Reddy et al. [21]	Single-center	UK	374	57.4	49.7	28.5
			407	55.8	46.4	28

**Table 4:** Intubation method used and side of lateral position.

Study		Supine vs. lateral positions	
		Intubation method used	Side of lateral position
Cheng et al. [22]	2004	Trach light	Lateral (right and left)
Dimitriou et al. [23]	2003	ILMA and flexible lightwand-guided intubation	Lateral (right and left)
Jin et al. [24]	2017	Video laryngoscopy (TIC-SC-II)	Left lateral
Khan et al. [5]	2010	Laryngoscopy	Left lateral
Komatsu et al. [25]	2011	Video laryngoscopy (Macintosh laryngoscope and airway scope)	Lateral (left and right)
Li et al. [26]	2016	Video laryngoscopy (flexible fiberoptic bronchoscope TIC-SCII, UE Medical Taizhou, Zhejiang, China)	Lateral
Naithani et al. [27]	2012	ILMA	Lateral
Naithani et al. [28]	2013	ILMA	Lateral
Panwar et al. [29]	2013	ILMA	Lateral (left and right)

**Table 5:** Intubation methods used and degree of semierect position.

Study		Supine vs. Semierect positions	
		Intubation method used	Degree of semierect position
Chang et al. [18]	2015	Direct laryngoscopy fiberoptic intubation (fiberoptic bronchoscope (LF-2; Olympus, Tokyo, Japan))	25° semisitting position
Collins et al. [19]	2004	Video laryngoscopy laryngoscope and video Macintosh laryngoscope system (VMS)—Karl Storz Endoscopy—America Inc.	35° for sniff ramped: multiple folded blankets until EAM horizontal with sternal notch; degree not mentioned
Gupta and Russin [20]	2012	Video laryngoscopy (GlideScope)	Semierect angle most comfortable for the patient
Reddy et al. [21]	2016	Laryngoscopy	25° sniffing position (semierect/back-up position)

## Data Availability

The data used to support the findings of this study are available from the authors upon request.

## Additional Points

The review was not registered in a public database. Aside from the PRISMA, there are no additional amendments to the information and the protocol.
